# Effectiveness of a Smartphone App to Promote Physical Activity Among Persons With Type 2 Diabetes: Randomized Controlled Trial

**DOI:** 10.2196/53054

**Published:** 2024-03-21

**Authors:** Stephanie E Bonn, Madeleine Hummel, Giulia Peveri, Helén Eke, Christina Alexandrou, Rino Bellocco, Marie Löf, Ylva Trolle Lagerros

**Affiliations:** 1 Clinical Epidemiology Division, Department of Medicine Solna Karolinska Institutet Stockholm Sweden; 2 Department of Medical Epidemiology and Biostatistics Karolinska Institutet Stockholm Sweden; 3 Department of Clinical Sciences and Community Health University of Milan Milan Italy; 4 Department of Biosciences and Nutrition Karolinska Institutet Stockholm Sweden; 5 Department of Statistics and Quantitative Methods University of Milano-Bicocca Milan Italy; 6 Center for Obesity, Academic Specialist Center Stockholm Health Services Stockholm Sweden

**Keywords:** behavior change, exercise, intervention, mHealth, smartphone app, self-monitoring

## Abstract

**Background:**

Physical activity is well known to have beneficial effects on glycemic control and to reduce risk factors for cardiovascular disease in persons with type 2 diabetes. Yet, successful implementation of lifestyle interventions targeting physical activity in primary care has shown to be difficult. Smartphone apps may provide useful tools to support physical activity. The DiaCert app was specifically designed for integration into primary care and is an automated mobile health (mHealth) solution promoting daily walking.

**Objective:**

This study aimed to investigate the effect of a 3-month-long intervention promoting physical activity through the use of the DiaCert app among persons with type 2 diabetes in Sweden. Our primary objective was to assess the effect on moderate to vigorous physical activity (MVPA) at 3 months of follow-up. Our secondary objective was to assess the effect on MVPA at 6 months of follow-up and on BMI, waist circumference, hemoglobin A_1c_, blood lipids, and blood pressure at 3 and 6 months of follow-up.

**Methods:**

We recruited men and women with type 2 diabetes from 5 primary health care centers and 1 specialized center. Participants were randomized 1:1 to the intervention or control group. The intervention group was administered standard care and access to the DiaCert app at baseline and 3 months onward. The control group received standard care only. Outcomes of objectively measured physical activity using accelerometers, BMI, waist circumference, biomarkers, and blood pressure were assessed at baseline and follow-ups. Linear mixed models were used to assess differences in outcomes between the groups.

**Results:**

A total of 181 study participants, 65.7% (119/181) men and 34.3% (62/181) women, were recruited into the study and randomized to the intervention (n=93) or control group (n=88). The participants’ mean age and BMI were 60.0 (SD 11.4) years and 30.4 (SD 5.3) kg/m^2^, respectively. We found no significant effect of the intervention (group by time interaction) on MVPA at either the 3-month (β=1.51, 95% CI –5.53 to 8.55) or the 6-month (β=–3.53, 95% CI –10.97 to 3.92) follow-up. We found no effect on any of the secondary outcomes at follow-ups, except for a significant effect on BMI at 6 months (β=0.52, 95% CI 0.20 to 0.84). However, mean BMI did not differ between the groups at the 6-month follow-up.

**Conclusions:**

We found no evidence that persons with type 2 diabetes being randomized to use an app promoting daily walking increased their levels of MVPA at 3 or 6 months’ follow-up compared with controls receiving standard care. The effect of the app on BMI was unclear, and we found nothing to support an effect on secondary outcomes. Further research is needed to determine what type of mHealth intervention could be effective to increase physical activity among persons with type 2 diabetes.

**Trial Registration:**

ClinicalTrials.gov NCT03053336; https://clinicaltrials.gov/study/NCT03053336

## Introduction

Despite increased knowledge and public health initiatives, more than 460 million people, corresponding to over 6% of the world’s population, are estimated to be diagnosed with type 2 diabetes today [1]. Persons with type 2 diabetes followed in primary care can be prescribed lifestyle interventions in combination with medications. Lifestyle interventions may include weight management, smoking cessation, stress reduction, and improved dietary habits or physical activity [2]. Physical activity is well known to have beneficial effects on glycemic control and to reduce risk factors for cardiovascular disease [3]. Yet, it has proven difficult to implement lifestyle interventions targeting physical activity in primary care [4]. Nevertheless, walking has been put forward as a useful therapeutic tool shown to improve glucose control, with clinically beneficial effects on blood glucose levels over time, and the potential to improve other clinical variables such as BMI and blood pressure in persons with type 2 diabetes [5].

During the past few decades, various telemonitoring, eHealth, and mobile health (mHealth) solutions targeting physical activity have been developed. Such solutions offer adaptable platforms for the delivery of self-management interventions that are easily accessible to both patients and health care practitioners, and users can engage with health information technology at their convenience. Smartphone apps may be useful in a health care setting to provide an additional tool to increase patients’ engagement through the use of self-monitoring of, for example, physical activity between routine visits [6].

Today, there are many commercially developed and available smartphone apps targeting self-management of chronic conditions. Common features of available apps targeted toward persons with type 2 diabetes include self-tracking of blood glucose levels and components targeting physical activity or diet in different ways [7,8]. Nevertheless, there is a wide variety in type and number of features for diabetes management in available apps [7], making it difficult for patients to select the most appropriate one to use. There are many commercial apps targeting lifestyle among persons with type 2 diabetes; however, few solutions primarily target physical activity within this group, and even fewer have been developed specifically for implementation in primary care. Therefore, we developed a digital platform and a smartphone app specifically for targeting physical activity among persons with type 2 diabetes treated within primary care [9]. The app was built to be integrated into the existing digital infrastructure of primary care in Sweden, with the aim to provide care givers and patients with a scientifically evaluated self-care management tool.

Results from studies evaluating the effectiveness of mHealth solutions, including smartphone apps, targeting persons with type 2 diabetes, have been summarized previously [10-15]. Significant reductions in hemoglobin A_1c_ (HbA_1c_) levels are generally shown after 3 months of follow-up. Most of the evaluated apps allowed the user to monitor their blood glucose levels and included physical activity or diet, either alone or in combination, as additional features. mHealth interventions targeting physical activity in adults in the general population have been shown to increase both minutes of physical activity and steps per day [16-18]. Nevertheless, apps primarily targeting physical activity, without including a component of glucose monitoring, in persons with type 2 diabetes are relatively uncommon. Poppe et al [19] evaluated a self-regulation–based eHealth and mHealth intervention primarily targeting physical activity in persons with type 2 diabetes and found positive results of the intervention on increased activity and decreased sedentary behavior, whereas Thorsen et al [20] found no effect of app-based interval walking on MVPA over 52 weeks compared with standard care. In summary, there is still a need to develop interventions targeting physical activity that are effective and can be implemented into primary care.

The aim of this study was to evaluate the effects of the DiaCert smartphone app promoting daily walking on moderate to vigorous physical activity (MVPA) and clinical variables in persons with type 2 diabetes. Our primary aim was to test the hypothesis that the app would lead to an increase in minutes of MVPA at 3 months compared with standard care only. Our secondary aim was to assess the effect of the app on MVPA after 6 months and on the clinical variables BMI, waist circumference, HbA_1c_, cholesterol (total, low-density lipoprotein [LDL], and high-density lipoprotein [HDL]), triglycerides, and systolic and diastolic blood pressure at 3 and 6 months. We hypothesized that the app would lead to improvements in both MVPA and clinical variables.

## Methods

### Trial Design

We conducted a randomized controlled trial with 2 parallel groups between February 2017 and June 2019. The DiaCert study design [21] has been described in detail previously. Study participants were randomized 1:1 to the intervention or control group at baseline. The primary study outcome was MVPA (minutes/day) at 3 months of follow-up. Secondary outcomes included MVPA at 6 months of follow-up and the clinical variables BMI, waist circumference, HbA_1c_, cholesterol (total, LDL, and HDL), triglycerides, and systolic and diastolic blood pressure at 3 and 6 months of follow-up. No changes to methods were done after trial commencement. The study is reported according to the CONSORT (Consolidated Standards of Reporting Trials) statement [22] and the CONSORT-EHEALTH (Consolidated Standards of Reporting Trials of Electronic and Mobile HEalth Applications and onLine TeleHealth) checklist, which is developed for eHealth or mHealth interventions [23].

### Ethical Considerations

The trial was approved by the ethics committee of the regional ethical review board in Stockholm, Sweden (Dnr: 2016/2041-31/2, 2016/99-32, 2017/1406-32) and registered at ClinicalTrials.gov (NCT03053336). All participants received both oral and written information about the study and provided their written informed consent to participate. Participants received no compensation for participation in the study. After data collection, data were anonymized.

### Study Participants

The inclusion criteria were (1) having a diagnosis of type 2 diabetes, (2) being 18 years of age or older, (3) being able to communicate in Swedish, and (4) having access to and being able to use a smartphone. The exclusion criterion was not being able to walk.

Patients were recruited continuously from 5 primary health care centers and 1 specialized medical center in the Stockholm area, Sweden. Patients at the participating centers received initial information about the study from their physician or diabetes nurse, and those interested in participating were contacted by study personnel and given more detailed information. Thereafter, patients either agreed to participate and were scheduled for a baseline introductory meeting, or declined participation. We did not record the number of patients who did not agree to participate. All study participants met with study personnel at baseline and after 3 and 6 months. On each occasion, study outcomes including physical activity and other lifestyle factors, as well as clinical variables, were assessed.

### Interventions

Study participants randomized to the intervention group continued to receive standard care as usual but also downloaded the DiaCert app during the baseline meeting. To access the individual user account in the app, a personal 6-digit code was entered. The code was given to participants by study personnel 7 days after the baseline meeting to avoid overlap with baseline accelerometer measurements. The intervention group was encouraged to use the app daily for 3 months. At the 3-month follow-up meeting, participants deleted the app from their phones together with study personnel. Participants then received no intervention during 3 months and were offered access to the app again at the 6-month follow-up.

The DiaCert app displayed daily steps that through connection to a digital platform were shared with study personnel. An individual step goal between 1000 and 10,000 steps [24] was set at baseline based on the participant’s usual activity level. Participants in the intervention group were contacted by study personnel every second week by phone. During these follow-ups, the participant could revise his or her step goal with an even 500 steps increase or decrease. The maximum goal set at any time point was 10,000 steps. Users received automatic positive feedback messages including the user’s name in the app on days when the goal was met. In addition to daily steps, information on HbA_1c_ taken during the study period was also displayed in the app.

The app design has previously been described in detail, including a presentation of the app screen by screen [9]. In brief, features of the app included a home screen displaying the daily steps in relation to an individual step goal during the past week. A circle was gradually filled as the user walked toward the step goal. It was completely filled and marked with a checkmark when the goal was reached. Through the home screen, the user was also able to access information on previous daily steps, questionnaires, and results of HbA_1c_. The app was continuously updated to run with the current iOS and Android versions, but no changes were made to the content during the study. Users were asked to contact study personnel if they experienced malfunction of the app. The app was developed within the research project and is no longer available.

Study participants randomized to the control group received standard care, that is, continued their usual care as prescribed by their regular primary care physician and diabetes nurse, also after inclusion to the study. For ethical reasons, they were offered access to the DiaCert app at the 6-month follow-up.

### Outcomes

#### Physical Activity

To assess the primary outcome of MVPA in this trial, physical activity was measured using the ActiGraph wGT3x-BT triaxial accelerometer (dynamic range: 8g) during 7 consecutive days. At each study meeting, the participants were asked to wear the accelerometer on their nondominant wrist day and night, starting at 4 PM the same day until 8 AM just over a week later. Participants wore the accelerometers on their wrist to increase feasibility and maximize compliance. Data were sampled at a frequency of 80 Hz.

We downloaded the collected data from each accelerometer using the manufacturer’s program (ActiLife Software, version 6.13.3; ActiGraph), and thereafter, the raw data were extracted for data processing. As suggested by Migueles et al [25], processing of accelerometer data was performed using the open-source R package GGIR. GGIR version 2.0-0, R version 3.6.1, and RStudio version 1.2.5019 were used. Data collected before the first and after the last midnight were excluded in order to examine 7 complete days. As the first step of analysis, data were averaged over 5-second epochs and aggregated through application of Euclidean norm minus 1, with negative values rounded up to 0. Autocalibration was performed using local gravity to adjust for calibration errors and unreliable signals.

The default cut point for MVPA (100 milligravity) and default settings for the definition and management of nonwear time (ie, 4×15 minutes) in GGIR were applied [26]. Nonwear time was by default replaced with imputed averaged activity from the same time the other measured days. A valid day was defined as at least 16 hours of wear time and a valid week required 4 valid days (including at least 1 weekend day) [25]. Variables were weighted 5:2 with data collected on weekdays and weekend days. For MVPA, bouts of consistent activity lasting for at least 1 minute were used, where 80% of the included epochs had to be above or equal to the cut point [27].

In addition to accelerometer measurements, we also assessed daily physical activity at baseline with 2 validated general questions used in routine health care [28]. Participants were asked to (1) report their usual time spent exercising during a week and (2) add up and report the total time estimated spent doing other types of leisure time physical activities of lower intensity in bouts of at least 10 minutes during a week. Walking, cycling, or gardening was presented to the respondent to exemplify activity level.

#### Clinical Variables

A detailed description of measurements has been published elsewhere [21]. In brief, HbA_1c_ (mmol/mol), total cholesterol (mmol/L), LDL cholesterol (mmol/L), HDL cholesterol (mmol/L), and triglycerides (mmol/L) were measured in fasting blood samples. HbA_1c_ was measured using the reference measurement procedure by the International Federation for Clinical Chemistry and Laboratory Medicine [29]. The enzymatic method [30] was used to measure total cholesterol and HDL cholesterol. LDL cholesterol was calculated using the Friedewald equation [31].

Height (to the nearest 0.5 cm), body weight (to the nearest 0.1 kg), and waist circumference (to the nearest 1.0 cm) were measured by study personnel who also performed 1 manual assessment of blood pressure (systolic and diastolic) after the participant had been sitting down for at least 5 minutes. BMI was calculated based on measured height and weight (kg/m^2^).

### Sample Size

Power calculations were performed a priori to determine the sample size need to detect a clinically significant difference of 8 minutes/day of MVPA [21]. A total of 250 participants (125 in each group) were estimated to provide 80% power at a 5% significance level. This included an expected dropout rate of 20%. Baseline data collection ended in June 2018 before reaching 250 participants because the DiaCert app was no longer compatible with the upgrades of iOS and Android.

### Randomization and Blinding

A random allocation sequence list was generated by the first author (SEB) in Stata (version 14.0; StataCorp). Women and men were randomized separately in blocks of 10 within each participating primary health care center and the specialized medical center. Patients who agreed to participate in the study were continuously allocated to the next available spot on the list by study personnel (authors SEB and CA). Participants were informed about their group allocation at the end of the baseline meeting. Because of the nature of the intervention, neither participants nor study personnel were blinded to participants’ group allocation.

### Statistical Methods

Baseline characteristics of study participants are presented as mean (SD) or number (%) for continuous and categorical variables, respectively. Variables were checked for normality and outliers. The Student *t* test or the chi-square test was used to assess potential differences in baseline characteristics between the intervention and control group.

We used linear mixed models with fixed and random intercept and slope for the time variables to assess if there were longitudinal differences in MVPA at 3 months of follow-up (primary outcome) and secondary outcomes including MVPA at 6 months of follow-up and BMI, waist circumference, HbA_1c_, total cholesterol, LDL cholesterol, HDL cholesterol, triglycerides, and systolic and diastolic blood pressure at 3 and 6 months of follow-up between the intervention and control group. We have therefore included, in addition to time and group terms, a group×time interaction term to assess if any differences in the study outcomes were constant at 3 and 6 months. For outcomes that differed significantly between the study groups at baseline, that is, MVPA, additional sensitivity analysis adjusting the models for baseline levels of the outcome was performed according to the methods described by Twisk et al [32]. The analysis of intervention effect was made following the intention-to-treat approach [33]. Missing data were associated with a primary care center, with 17/25 (68%) participants with missing accelerometer data at baseline belonging to primary care center 1. Missing data were assumed to be missing at random and not depending on the study group as the degree of missing data was similar in both the intervention and control group during the intervention. Participants with complete data at baseline for each specific outcome variable were included in the analysis of intervention effect at 3 and 6 months.

Post hoc sensitivity analyses using self-reported data on physical activity from the baseline questionnaire were carried out to deal with the unblinded nature of our study and the potential bias that may have been present during baseline accelerometer measurements. Although participants were not connected to the DiaCert app until after the completion of baseline accelerometer measurements, they were aware of which group they were randomized to during measurements. This could potentially have affected baseline levels of MVPA, which might not have represented the “true” levels of MVPA before the start of the study. Multiple imputation based on MICE (multiple imputation by chained equations) was used to address this issue [34]. We first set all baseline values of MVPA for the intervention group to missing. To predict MVPA at baseline for participants in the intervention group in the hypothetical scenario in which participants were blind to the intervention, we implemented MICE using all relevant baseline variables. The variables included in the model were age, sex, height, weight, education, household income, marital status, smoking and snuff habits, the year of diabetes diagnosis, the center of recruitment, self-reported levels of physical activity, and the number of valid accelerometer days at baseline. We compared the imputed and the observed values of MVPA at baseline using a 2-tailed *t* test. Linear mixed models, as described above, were thereafter fitted to contrast differences in MVPA between the intervention and control group using the imputed data for MVPA at baseline.

Statistical tests were 2-sided, and the significance level was set to *P*<.05. Statistical analyses were performed using Stata (version 17; StataCorp).

## Results

**Figure 1:**
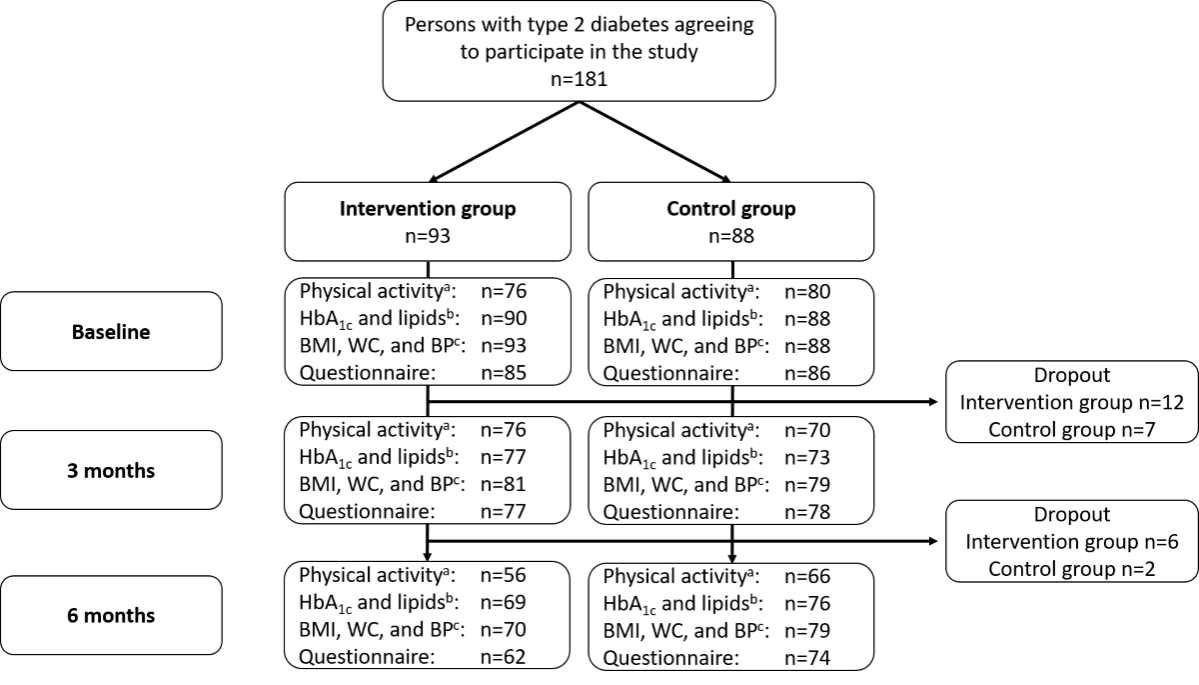
Flowchart of participation and data completeness at baseline and follow-up after 3 and 6 months in the DiaCert study. ^a^Number of participants with valid accelerometer data, that is, at least 16 hours of wear time and a total of 4 valid days including at least 1 weekend day; ^b^Triglycerides and cholesterol (total, low-density lipoprotein, and high-density lipoprotein). BP: blood pressure; HbA_1c_: hemoglobin A_1c_; WC: waist circumference.

A total of 181 persons with type 2 diabetes were included in the trial, of whom 93 were randomized to the intervention group and 88 were randomized to the control group. At 3 months, the dropout rate was 10.5% (19/181), and at 6 months it was 14.9% (27/181). Dropout was higher among participants in the intervention group (n=12 vs n=7 at 3 months, and n=6 vs n=2 at 6 months) than among those in the control group. In total, 156 participants had valid accelerometer data on physical activity at baseline. Of these, 137 (87.8%) also had valid data at the 3-month follow-up (primary outcome). At baseline, most participants (75.6%, 118/156) had valid accelerometer data from all 7 days, 14.7% (23/156) had valid data from 6 days, and 9.6% (15/156) from 5 or 4 days. A flowchart of participants with complete data from the different assessments is presented in Figure 1.

Characteristics of all 181 study participants are presented in Table 1. The majority of participants were men (119/181, 65.8%), and the mean (SD) age at baseline among all participants was 60.0 (11.4) years. The mean (SD) BMI among all participants was 30.4 (5.3) kg/m^2^. Overweight and obesity was common, 85.6% (155/181) had a BMI of ≥25 kg/m^2^, and 48.1% (87/181) had a BMI of ≥30 kg/m^2^. Mean (SD) HbA_1c_ was 53.6 (12.8) mmol/mol. There were no statistically significant differences between the study groups regarding age distribution, primary care center belonging, sex, smoking, time since diabetes diagnosis, or clinical variables including HbA_1c_ and lipid levels. In study participants with complete accelerometer data at baseline (n=156), there was a statistically significant difference in accelerometer-measured baseline levels of physical activity between the intervention and control group, with higher MVPA (38.3 vs 29.8 minutes/day, *P*=.04) measured in the intervention group. Using the imputed data for the intervention group, the baseline MVPA was estimated to be 32.3 minutes/day, which did not differ from the measured level in the control group (*P*=.62). Additionally, self-reported levels of physical activity at baseline, that is, time spent exercising and total leisure time activity, did not differ between the groups (*P*=.20 and *P*=.20, respectively). Baseline characteristics of participants with complete accelerometer data at baseline can be found in Multimedia Appendix 1.

**Table 1:** Characteristics of study participants by study group (N=181).

Characteristic			Intervention group (n=93)				Control group (n=88)					*P* value^a^	
			n (%)		Mean (SD)		n (%)		Mean (SD)				
MVPA^b,c^ (minutes/day)			76 (82)		38.3 (28.3)		80 (91)		29.7 (24.1)		*.04*		
BMI (kg/m^2^)			93 (100)		30.2 (5.5)		88 (100)		30.6 (5.2)		.61		
**Waist circumference**													
	Women	33 (35)		102 (12.6)		28 (32)		103 (15.8)		.75			
	Men	59 (63)		111 (15.6)		60 (68)		110 (13.3)		.75			
Hemoglobin A_1c_ (mmol/mol)			89 (96)		53.6 (13.0)		88 (100)		53.5 (12.7)		.97		
Total cholesterol (mmol/L)			74 (80)		4.56 (1.00)		70 (80)		4.50 (1.18)		.77		
LDL^d^ cholesterol (mmol/L)			73 (78)		2.70 (0.89)		70 (80)		2.64 (1.13)		.72		
HDL^e^ cholesterol (mmol/L)			74 (80)		1.25 (0.34)		70 (80)		1.23 (0.40)		.73		
Triglycerides (mmol/L)			72 (77)		1.54 (0.76)		69 (78)		1.64 (0.94)		.45		
**Blood pressure (mm Hg)**													
	Systolic	92 (99)		138.7 (16.2)		88 (100)		137.0 (14.8)		.45			
	Diastolic	92 (99)		83.6 (9.9)		88 (100)		82.6 (9.5)		.50			
**Sex**													.50
	Male	34 (37)		—^f^		28 (32)		—					
	Female	59 (63)		—		60 (68)		—					
**Age (years)**													.24
	<50	20 (22)		—		16 (18)		—					
	50-59	28 (30)		—		19 (22)		—					
	60-69	31 (33)		—		30 (34)		—					
	≥70	14 (15)		—		23 (26)		—					
**Leisure time activity^g^ (minutes/week)**											.27		
	<60	6 (7)				11 (13)		—					
	60-90	8 (10)		—		8 (10)		—					
	90-150	17 (20)		—		15 (18)		—					
	150-300	14 (17)		—		22 (26)		—					
	>300	39 (46)		—		28 (33)		—					
**Primary care centers**													.88
	1	35 (38)		—		31 (35)		—					
	2	14 (15)		—		13 (15)		—					
	3	6 (6)		—		8 (9)		—					
	4	23 (25)		—		23 (26)		—					
	5	10 (11)		—		11 (13)		—					
	Specialized medical center	5 (5)		—		2 (2)		—					
**Time spent exercising^h^ (minutes/week)**													.26
	Never	35 (42)		—		38 (44)		—					
	<30	12 (14)		—		12 (14)		—					
	30-90	13 (15)		—		21 (24)		—					
	>90	24 (29)		—		15 (17)		—					
**Smoking^i^**													.41
	Yes	11 (13)		—		9 (11)		—					
	No, ever smoker	31 (37)		—		40 (47)		—					
	No, never smoker	42 (50)		—		36 (42)		—					
**Time since diabetes diagnosis^j^ (years)**													.50
	<1	8 (11)		—		13 (18)		—					
	1-5	20 (29)		—		17 (24)		—					
	>5	42 (60)		—		42 (58)		—					
**Education^k^ (years)**													.69
	≤12	45 (54)		—		44 (51)		—					
	>12	38 (46)		—		42 (49)		—					

^a^2-tailed *t* test was used for continuous variables and the chi-square test was used for categorical variables. Italicized *P* values represent statistical significance.

^b^MVPA: moderate to vigorous physical activity.

^c^Missing data from n=17 (intervention) and n=8 (control).

^d^LDL: low-density lipoprotein.

^e^HDL: high-density lipoprotein.

^f^—not available.

^g^From questionnaire, missing data n=9 (intervention) and n=4 (control).

^h^From questionnaire, missing data n=9 (intervention) and n=2 (control).

^i^Missing data n=9 (intervention) and n=3 (control).

^j^Missing data n=23 (intervention) and n=16 (control).

^k^Missing data n=10 (intervention) and n=2 (control).

### Effectiveness of the Intervention—MVPA

**Figure 2:**
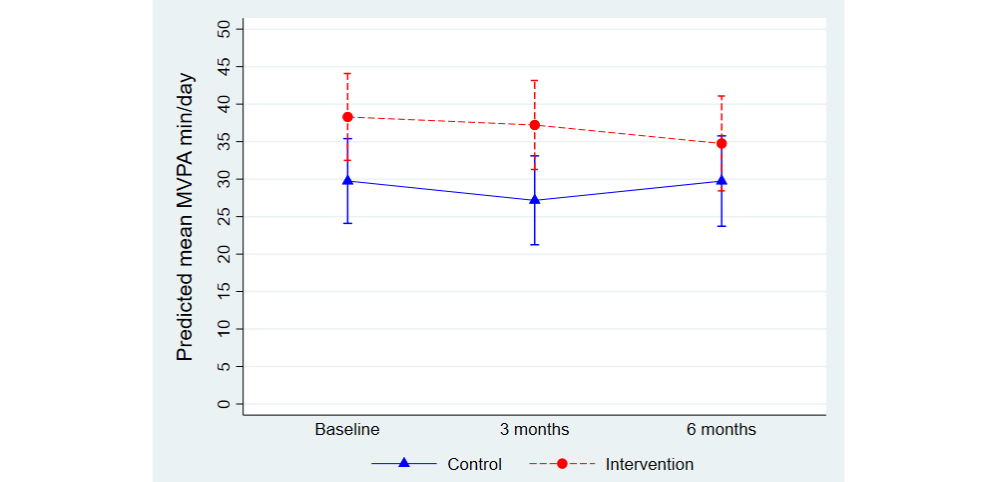
Changes over time in moderate to vigorous physical activity (MVPA, minutes/day) in the intervention and control group. Predicted group means from linear mixed model analysis. Results correspond to model estimates shown in Table 2.

Results from between-group analysis and the intervention effect on minutes/day of MVPA are shown in Table 2. The mean change in minutes/day of MVPA from baseline to follow-ups is graphically shown in Figure 2. The statistically significant difference in minutes/day of MVPA seen between the groups at baseline, with participants in the intervention group being more active than participants in the control group, remained at the 3-month follow-up. The predicted mean difference between the groups after 3 months was 10.05 minutes (95% CI 1.66-18.44). At the 6-month follow-up, there was no statistically significant difference in MVPA between the groups (β=5.02, 95% CI –3.72 to 13.75).

**Table 2:** The intervention effect on the primary outcome of daily minutes of moderate to vigorous physical activity (MVPA) at 3 months of follow-up and on secondary outcomes including MVPA at 6 months of follow-up and clinical variables in the DiaCert study.

Characteristic			Group sample means											Model estimates^a^			
			Intervention (n=93)					Control (n=88)					Difference^b^				Group by time interaction
			n (%)		Mean (SD)		n (%)			Mean (SD)		Mean (95% CI)				β (95% CI)	
**MVPA (minutes/day)**																	
	3 months	70 (75)		36.6 (25.5)		67 (76)			26.7 (21.1)		10.05 (1.66 to 18.44)				1.51 (–5.53 to 8.55)		
	6 months	55 (59)		34.2 (29.4)		63 (72)			31.1 (27.0)		5.02 (–3.72 to 13.75)				–3.53 (–10.97 to 3.92)		
**BMI (kg/m^2^)**																	
	Baseline	93 (100)		30.2 (5.5)		88 (100)			30.6 (5.2)		N/A^c^				N/A		
	3 months	81 (87)		30.1 (5.7)		79 (90)			30.3 (5.0)		–0.12 (–1.67 to 1.44)				0.29 (–0.02 to 0.61)		
	6 months	70 (75)		30.0 (6.0)		79 (90)			29.9 (5.0)		0.11 (–1.45 to 1.67)				0.52 (0.20 to 0.84)		
**Waist circumference (cm)**																	
	Baseline	92 (99)		107 (15.1)		88 (100)			108 (14.4)		N/A				N/A		
	3 months	80 (86)		107 (15.6)		79 (90)			108 (13.6)		–0.61 (–4.90 to 3.69)				–0.46 (–1.75 to 0.83)		
	6 months	69 (74)		107 (17.0)		79 (90)			106 (13.6)		0.47 (–3.84 to 4.77)				0.61 (–0.71 to 1.94)		
**Hemoglobin A_1c_ (mmol/mol)**																	
	Baseline	89 (96)		53.6 (13.0)		88 (100)			53.5 (12.7)		N/A				N/A		
	3 months	75 (81)		50.0 (9.9)		73 (83)			53.2 (13.4)		–2.45 (–6.08 to 1.17)				–2.54 (–5.36 to 0.29)		
	6 months	67 (72)		51.2 (10.7)		76 (86)			51.2 (10.6)		–0.21 (–3.87 to 3.44)				–0.30 (–3.16 to 2.57)		
**Total cholesterol (mmol/L)**																	
	Baseline	74 (80)		4.56 (1.00)		70 (80)			4.50 (1.18)		N/A				N/A		
	3 months	62 (67)		4.39 (0.80)		57 (65)			4.48 (0.91)		–0.06 (–0.40 to 0.28)				–0.11 (–0.37 to 0.14)		
	6 months	55 (59)		4.38 (0.84)		59 (67)			4.27 (1.02)		0.10 (–0.24 to 0.44)				0.05 (–0.21 to 0.31)		
**LDL^d^ cholesterol (mmol/L)**																	
	Baseline	73 (78)		2.70 (0.89)		70 (80)			2.64 (1.13)		N/A				N/A		
	3 months	59 (63)		2.44 (0.65)		56 (64)			2.48 (0.89)		–0.01 (–0.33 to 0.30)				–0.07 (–0.30 to 0.15)		
	6 months	54 (58)		2.44 (0.68)		57 (65)			2.38 (0.85)		0.09 (–0.23 to 0.40)				0.02 (–0.20 to 0.25)		
**HDL^e^ cholesterol (mmol/L)**																	
	Baseline	74 (80)		1.25 (0.34)		70 (80)			1.23 (0.40)		N/A				N/A		
	3 months	62 (67)		1.22 (0.34)		57 (65)			1.24 (0.38)		–0.04 (–0.16 to 0.09)				–0.06 (–0.12 to 0.01)		
	6 months	55 (59)		1.26 (0.36)		57 (65)			1.24 (0.39)		–0.04 (–0.17 to 0.08)				–0.06 (–0.13 to 0.001)		
**Triglycerides (mmol/L)**																	
	Baseline	72 (77)		1.54 (0.76)		69 (78)			1.65 (0.94)		N/A				N/A		
	3 months	60 (65)		1.71 (1.03)		58 (66)			1.76 (0.96)		–0.01 (–0.32 to 0.29)				0.10 (–0.15 to 0.34)		
	6 months	52 (56)		1.56 (0.83)		56 (64)			1.66 (0.83)		–0.07 (–0.38 to 0.24)				0.04 (–0.21 to 0.29)		
**Systolic BP^f^ (mm Hg)**																	
	Baseline	92 (99)		139 (16.2)		88 (100)			137 (14.8)		N/A				N/A		
	3 months	80 (86)		135 (13.3)		79 (90)			134 (11.8)		1.21 (–2.98 to 5.40)				–0.54 (–4.62 to 3.53)		
	6 months	70 (75)		136 (12.8)		78 (89)			136 (12.6)		0.70 (–3.60 to 4.99)				–1.05 (–5.23 to 3.13)		
**Diastolic BP** **(mm Hg)**																	
	Baseline	92 (99)		83.6 (9.9)		88 (100)			82.6 (9. 5)		N/A				N/A		
	3 months	80 (86)		81.8 (9.1)		79 (90)			79.2 (10.5)		2.78 (–0.14 to 5.69)				1.79 (–1.06 to 4.64)		
	6 months	70 (75)		81.2 (8.7)		78 (89)			79.9 (9.4)		1.87 (–1.12 to 4.85)				0.88 (–2.04 to 3.81)		

^a^Results from linear mixed model analysis including participants with complete baseline data.

^b^Difference between groups at the specified time point based on predicted means from linear mixed models.

^c^—: not applicable.

^d^LDL: low-density lipoprotein.

^e^HDL: high-density lipoprotein.

^f^BP: blood pressure.

We found no statistically significant effect of the intervention (group by time interaction) on MVPA at either the 3- or the 6-month follow-up (Table 2). When adjusting for baseline levels of MVPA, results remained nonsignificant at both 3 (β=4.38, 95% CI –2.11 to 10.88) and 6 (β=–0.65, 95% CI –7.58 to 6.29) months. Additionally, results from sensitivity analyses using imputed baseline data also remained nonsignificant at follow-up after both 3 (β=6.86, 95% CI –4.05 to 17.78) and 6 (β=1.44, 95% CI –9.87 to 11.76) months.

### Effectiveness of Intervention—Clinical Variables

Detailed results from between-group analyses and the intervention effect on clinical variables included as secondary outcomes are shown in Table 2. The mean change in outcomes from baseline to follow-ups is graphically shown in Multimedia Appendix 2. We found no statistically significant differences in any of the secondary outcomes at the 3- or 6-month follow-ups, except for in the analysis of BMI where a statistically significant effect of the intervention was seen at 6 months (group by time interaction: 0.52, 95% CI 0.20-0.84). However, there was no difference in mean BMI between the groups at the 6-month follow-up (predicted difference in mean: 0.11, 95% CI –1.45 to 1.67). Participants in the control group had a slightly higher BMI at baseline than participants in the intervention group (30.6 vs 30.2 kg/m^2^), although this difference was not statistically significant (*P*=.61).

## Discussion

### Principal Findings

In this 2-armed randomized controlled trial, we found no clear effect of the DiaCert app promoting daily walking in persons with type 2 diabetes. We found no increase in objectively measured MVPA after neither 3 (primary outcome) nor 6 months of follow-up compared with standard care when accounting for baseline levels of MVPA. This is in line with results from Thorsen et al [20]. They were not able to show an effect of app-based interval walking on MVPA over 52 weeks compared with standard care among persons with type 2 diabetes. On the contrary, Poppe et al [19] found that an eHealth and mHealth intervention that primarily targeted physical activity in persons with type 2 diabetes led to increased physical activity and decreased sedentary behavior. Participants in our study and the study by Thorsen et al [20] reported more time in MVPA at baseline compared with those in the study by Poppe et al [19]. This might have contributed to the difference in effect between the studies, as a higher baseline level of MVPA may imply less room for improvement.

Other previous studies have also shown an effect of mHealth solutions to increase physical activity. Hochsmann et al [35] showed that an interactive smartphone game aimed at increasing daily steps in persons with type 2 diabetes had a significant effect on activity compared with a control group receiving standard lifestyle counseling. Glynn et al [36] evaluated the effect of an app that aimed to increase physical activity in primary care patients and found that the intervention group increased their number of daily steps compared with the control group. In the randomized Sophia step study, comprising persons with prediabetes or type 2 diabetes in Sweden [37], self-monitoring of daily physical activity using pedometers with registration of steps on the web in combination with counseling did not increase levels of physical activity but seemed to prevent the decrease in physical activity seen in the control group. Although our primary hypothesis was rejected and we could not show that the use of the DiaCert app led to increased MVPA among persons with type 2 diabetes, results from other studies still indicate that mHealth solutions can have positive effects on physical activity.

Although we found a statistically significant effect of the DiaCert intervention on BMI at the 6-month follow-up, there was no difference in mean BMI between the groups at any time point. Therefore, we cannot draw any conclusion regarding the effect on BMI. Not all studies evaluating interventions targeting physical activity include anthropometric outcomes, and the effect on BMI in previous studies are mixed, showing no effect or indicating a small reduction favoring the intervention group [10,12]. Thorsen et al [20] did not report BMI as an outcome, but found a nonsignificant reduction of waist circumference. Similar to the Sophia step study, our results provided no evidence for an effect on waist circumference [37].

Reviews and meta-analyses [11,13,15] provide strong evidence for a positive effect of mobile apps for lifestyle modification in persons with type 2 diabetes on HbA_1c_ levels. However, all of the evaluated apps included monitoring of blood glucose. The effects on other clinical markers including body weight, blood lipids, and blood pressure are less clear [38]. Our results are in line with the Sophia step study, where no effects on cardiometabolic variables, including HbA_1c_, triglycerides, HDL cholesterol, and LDL cholesterol, were seen [37]. Neither our study nor the Sophia step study included a glucose-monitoring component, which may explain the lack of an effect on HbA_1c_. Nevertheless, results from a meta-analysis by Lee et al [12] indicated that mHealth interventions in persons with type 2 diabetes >65 years of age may improve blood lipid profiles, which we found no evidence of. One explanation for the lack of an effect on cardiometabolic markers is that a changed lifestyle behavior for the better could have led to lowered medication, leaving HbA_1c_, serum lipid levels, or blood pressure unchanged. We did not assess changes in medications during the intervention, which is a limitation of our study.

The use of different behavior change techniques may also explain differences in efficacy between apps. Fanning et al [39] investigated the effect of a basic self-monitoring app (tracking, feedback, information) and 2 theory-based tools (goal setting and point-based feedback) on MVPA among healthy but inactive adults. Four different groups received either a basic self-monitoring app only or the basic app together with (1) goal setting, or (2) feedback, or (3) goal setting and feedback. All groups increased their MVPA, but the feedback group showed the highest increase. While the DiaCert app comprised several behavioral change techniques that previously have been included in successful interventions, such as monitoring, goal setting, and positive feedback, it did not include other features associated with effective results, for example, frequent reminders or the option to share data with peers [40]. How the included components are designed may also affect results; for example, goal setting can be personalized or generic, and a goal can be more or less challenging.

### Strengths and Limitations

The design of this randomized controlled study is one of the strengths of our study. The fact that study participants were recruited from 6 different care centers located in different areas with diverse populations and levels of socioeconomic status is another strength. This likely increased generalizability of our results. However, a limitation of our study is that we did not record the number of patients who turned down participation after being contacted by study personnel. Nevertheless, the mean age in our study was slightly lower than that of the average person with type 2 diabetes in Sweden, but levels of BMI and HbA_1c_ were similar [41]. Younger persons may be more inclined to participate in an app-based intervention, although internet access and smartphone usage are high also among older age groups in Sweden [42]. Another strength of our study is that both men and women were included. Earlier studies have shown that women participate in physical activity programs more than men [43]. The larger proportion of men in our study could partly be explained by the higher prevalence of type 2 diabetes among men; almost 60% of persons with type 2 diabetes within primary care in Sweden are men [41]. It could also be speculated that older men are more interested in using technology than older women and therefore more likely to participate in an mHealth intervention.

The fact that participants were not blinded to the intervention is a limitation. While baseline information regarding the clinical variables is unlikely to have been affected by participants being aware of their group allocation, this knowledge may have had an impact on accelerometer measurements. Participants can, intentionally or unintentionally, change their physical activity behaviors during the measuring period. The statistically significant differences between the intervention and control group in accelerometer-measured MVPA at baseline were not seen for physical activity assessed in the baseline questionnaire. This could be interpreted as an immediate effect of being randomized to the intervention group and thereby feeling encouraged to become more active. Future studies should be careful not to disclose allocation information to participants until all baseline measures have been completed. It is also a limitation that personnel working in the study were not blinded during measurements. Nevertheless, objective accelerometer measurement of physical activity and clinical biomarkers including HbA_1c_ and lipid levels represent a strength in our study design as they are less prone to biased estimates resulting from unblinded study personnel. The continuous recruitment of study participants during the whole year also reduced the risk of results being biased due to season.

We did not reach our goal of including 250 participants in the study, which is a limitation. Because a digital solution must be continuously updated to run with the current iOS and Android versions, we had to end recruitment after 2.5 years for practical reasons, before reaching the goal. However, dropout rates were lower than the estimated 20% in our power calculation [21]. Lack of data on user engagement and adherence to the app is a limitation as higher user engagement has also been associated with more favorable outcomes [44]. Decreased app engagement over time during an intervention period has been suggested as a potential explanation for the lack of more long-term effects of physical activity promoting apps [45].

### Conclusions

We found no evidence that persons with type 2 diabetes being randomized to use an app promoting daily walking increased their levels of MVPA at 3 or 6 months of follow-up compared with controls receiving standard care. Further, the effect on BMI was unclear, and we found nothing to support an effect of being randomized to use the DiaCert app on waist circumference, HbA_1c_, total cholesterol, LDL cholesterol, HDL cholesterol, triglycerides, and systolic or diastolic blood pressure compared with standard care. Further research is needed to determine what type of mHealth physical activity intervention could be effective to increase physical activity and improve cardiometabolic markers among persons with type 2 diabetes.

## Appendix

Multimedia Appendix 1 Characteristics of study participants with complete accelerometer data at baseline (n=156) by study group.

Multimedia Appendix 2 Changes over time in secondary outcomes in the intervention and control group. Predicted group means from linear mixed model analysis; results correspond to model estimates shown in Table 2.

Multimedia Appendix 3 CONSORT eHEALTH checklist.

## Abbreviations

CONSORT: Consolidated Standards of Reporting Trials
CONSORT-EHEALTH: Consolidated Standards of Reporting Trials of Electronic and Mobile HEalth Applications and onLine TeleHealth
HbA_1c_: hemoglobin A_1c_
HDL: high-density lipoprotein
LDL: low-density lipoprotein
mHealth: mobile health
MICE: multiple imputation by chained equations
MVPA: moderate to vigorous physical activity

## Data Availability

The data sets generated during and analyzed during this study are not publicly available due ethical restrictions but are available from the corresponding author on reasonable request.
