# Supplementary material for: Effectiveness of a Smartphone App to Promote Physical Activity Among Persons With Type 2 Diabetes: Randomized Controlled Trial
Source: Interact J Med Res. 2024 Mar 21;13:e53054. doi: 10.2196/53054 (PMC10995783; doi:10.2196/53054)
Supplement: Multimedia Appendix 1 [file ijmr_v13i1e53054_app1.docx]

| **Multimedia Appendix 1.** Characteristics of study participants with complete accelerometer data at baseline (n=156) by study group. | | | | | | | | | |
| --- | --- | --- | --- | --- | --- | --- | --- | --- | --- |
|  | | | **Intervention group (n=76)** | | | **Control group (n=80)** | | | *P*^a^ |
|  | | | **n** | **mean** | **(SD)** | **n** | **mean** | **(SD)** |  |
| **MVPA**, min/day | | | 76 | 38.3 | (28.3) | 80 | 29.8 | (24.1) | **.04** |
| **BMI**, kg/m^2^ | | | 76 | 29.7 | (5.6) | 80 | 30.4 | (5.3) | .43 |
| **Waist circumference** | | |  |  |  |  |  |  |  |
|  | | Women | 25 | 101 | (12.9) | 25 | 103 | (17.1) | .60 |
|  | | Men | 50 | 110 | (15.7) | 56 | 110 | (13.3) | .90 |
| **HbA1c**, mmol/mol | | | 73 | 53.8 | (13.6) | 80 | 53.3 | (12.6) | .80 |
| **Total cholesterol**, mmol/L | | | 63 | 4.56 | (0.99) | 64 | 4.43 | (1.12) | .48 |
| **LDL-cholesterol**, mmol/L | | | 63 | 2.70 | (0.88) | 64 | 2.57 | (1.02) | .43 |
| **HDL-cholesterol**, mmol/L | | | 63 | 1.25 | (0.34) | 64 | 1.25 | (0.40) | .97 |
| **Triglycerides,** mmol/L | | | 61 | 1.49 | (0.78) | 63 | 1.55 | (0.87) | .70 |
| **Blood pressure**, mmHg | | |  |  |  |  |  |  |  |
|  | | Systolic | 76 | 138.0 | (16.9) | 80 | 136.3 | (14.2) | .49 |
|  | | Diastolic | 76 | 82.9 | (9.6) | 80 | 81.9 | (8.5) | .47 |
|  | | |  | **n** | **(%)** |  | **n** | **(%)** |  |
| **Sex** | | |  |  |  |  |  |  | .57 |
|  | Women | |  | 26 | (34.2) |  | 24 | (30.0) |  |
|  | Men | |  | 50 | (65.8) |  | 56 | (70.0) |  |
| **Age** | | |  |  |  |  |  |  | .22 |
|  | <50 years | |  | 15 | (19.7) |  | 13 | (16.3) |  |
|  | 50-59 years | |  | 23 | (30.3) |  | 16 | (20.0) |  |
|  | 60-69 years | |  | 26 | (34.2) |  | 29 | (36.3) |  |
|  | ≥70 years | |  | 12 | (15.8) |  | 22 | (27.5) |  |
| **Leisure time activity**^b^ | | |  |  |  |  |  |  | .20 |
|  | < 60 min/week | |  | 4 | (5.6) |  | 8 | (10.4) |  |
|  | 60 to 90 min/week | |  | 7 | (9.7) |  | 8 | (10.4) |  |
|  | 90 to 150 min/week | |  | 15 | (20.8) |  | 15 | (19.5) |  |
|  | 150 to 300 min/week | |  | 11 | (15.3) |  | 21 | (27.3) |  |
|  | > 300 min/week | |  | 35 | (48.6) |  | 25 | (32.5) |  |
| **Primary care centers** | | |  |  |  |  |  |  | .87 |
|  | 1 | |  | 22 | (29.0) |  | 27 | (33.8) |  |
|  | 2 | |  | 14 | (18.4) |  | 11 | (13.8) |  |
|  | 3 | |  | 5 | (6.6) |  | 7 | (8.8) |  |
|  | 4 | |  | 21 | (27.6) |  | 22 | (27.5) |  |
|  | 5 | |  | 10 | (13.2) |  | 11 | (13.8) |  |
|  | Specialized medical center | |  | 4 | (5.3) |  | 2 | (2.5) |  |
| **Time spent exercising**^b^ | | |  |  |  |  |  |  | .20 |
|  | Never | |  | 30 | (41.7) |  | 35 | (43.8) |  |
|  | < 30 min/week | |  | 11 | (15.3) |  | 11 | (13.8) |  |
|  | 30-90 min/week | |  | 11 | (15.3) |  | 20 | (25.0) |  |
|  | > 90 min/week | |  | 20 | (28.8) |  | 14 | (17.5) |  |
| **Smoking^c^** | | |  |  |  |  |  |  | .55 |
|  | Yes | |  | 9 | (11.8) |  | 8 | (10.0) |  |
|  | No, ever smoker | |  | 27 | (35.5) |  | 37 | (46.3) |  |
|  | No, never smoker | |  | 35 | (46.1) |  | 34 | (42.5) |  |
| **Time since diabetes diagnosis^d^** | | |  |  |  |  |  |  | .44 |
|  | <1 year | |  | 8 | (10.5) |  | 13 | (16.3) |  |
|  | 1 – 5 years | |  | 19 | (25.0) |  | 15 | (18.8) |  |
|  | >5 years | |  | 37 | (48.7) |  | 40 | (50.0) |  |
| **Education^e^** | | |  |  |  |  |  |  | .84 |
|  | ≤12 years | |  | 37 | (48.7) |  | 41 | (51.3) |  |
|  | >12 years | |  | 33 | (43.4) |  | 39 | (48.8) |  |
| ^a^t-test continuous and chi-2 categorical, ^b^From questionnaire, ^c^missing data n=5 (intervention) and n=1 (control), ^d^missing data n=12 (intervention) and n=12 (control), ^e^missing data n=6 (intervention) | | | | | | | | | |
