# Supplementary figures and images for: Effectiveness of a Smartphone App to Promote Physical Activity Among Persons With Type 2 Diabetes: Randomized Controlled Trial
Source: Interact J Med Res. 2024 Mar 21;13:e53054. doi: 10.2196/53054 (PMC10995783; doi:10.2196/53054)

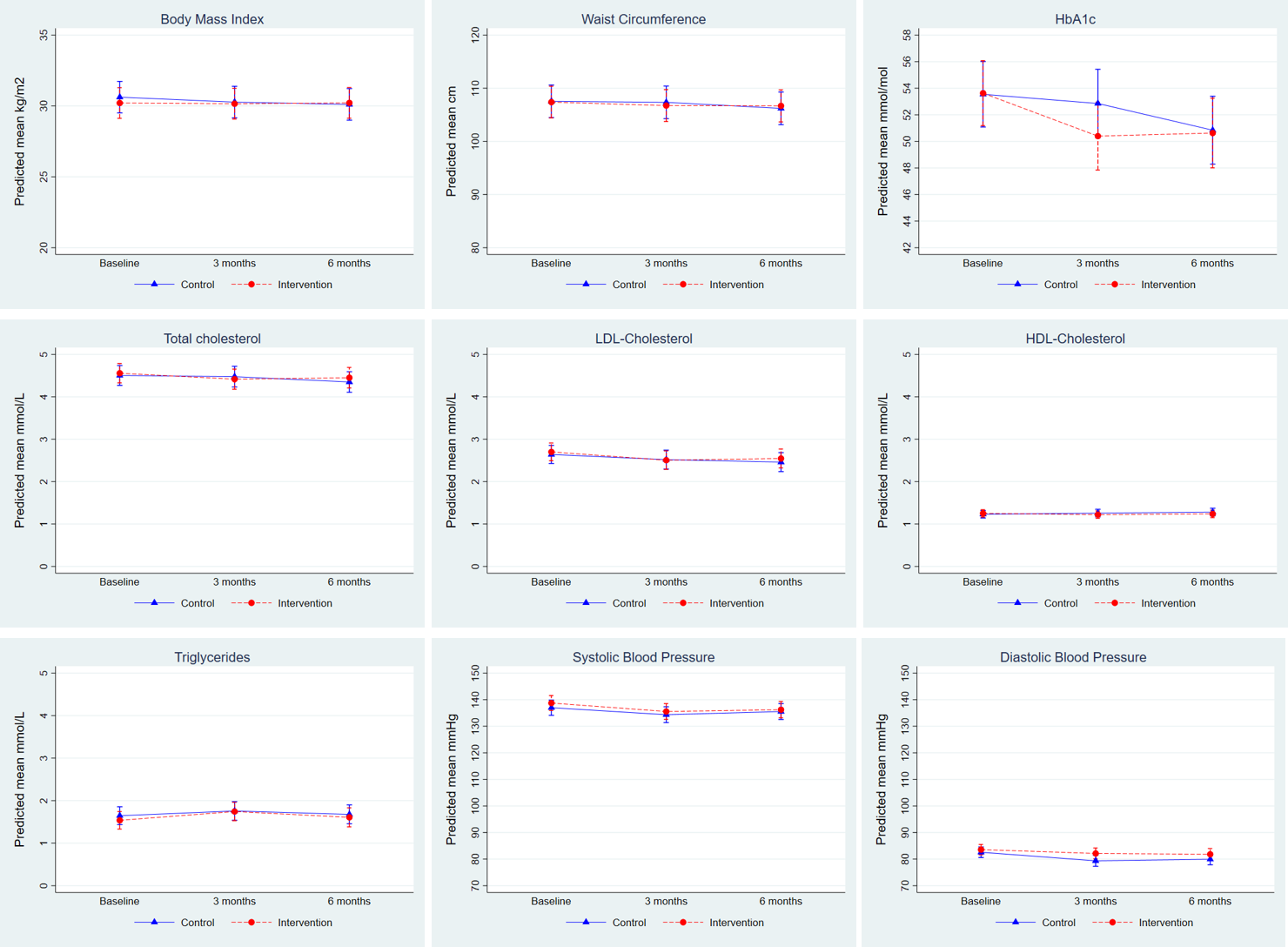

Supplement: Multimedia Appendix 2 [file ijmr_v13i1e53054_app2.png]
